# 小细胞肺癌免疫治疗及预后相关标志物的研究进展

**DOI:** 10.3779/j.issn.1009-3419.2020.03.08

**Published:** 2020-03-20

**Authors:** 青仪 王, 文颖 彭, 美林 江, 麟 邬

**Affiliations:** 410006 长沙，中南大学湘雅医学院附属肿瘤医院胸内二科 Second Department of Thoracic Oncology, Hunan Cancer Hospital and The Affiliated Cancer Hospital of Xiangya School of Medicine, Central South University, Changsha 410006, China

**Keywords:** 肺肿瘤, 免疫治疗, 生物标志物, Lung neoplsma, Immunotherapy, Predictive marker

## Abstract

近年来，虽然非小细胞肺癌患者的治疗效果得到了明显的改善，但小细胞肺癌（small cell lung cancer, SCLC）患者仍因治疗选择有限而预后很差。SCLC发病率占肺癌总体发病率的15%，具有恶性程度高、生长迅速、易发生远处转移及复发的特点，治疗十分棘手。随着免疫治疗获批于非小细胞肺癌等多种实体瘤，作为免疫源性相对较强的癌种，SCLC的相关临床研究也在开展中，并已取得一定的进展。另一方面，由于肿瘤异质性的存在，探索能够预测SCLC疗效的相关标志物对患者精准治疗至关重要。本综述阐述了目前SCLC免疫治疗及与SCLC免疫治疗相关生物标志物研究的最新进展。

## Abstract (en)

The efficacy of non-small cell lung cancer (NSCLC) has been obviously improved recent years, while the survival of small cell lung cancer (SCLC) patients remains inferior for limit treatment options. The incidence of SCLC accounts for 15% of the overall incidence of lung cancer, and it is characterized with high malignancy, rapid growth, early widespread metastasis, making it very difficult to treat. With the approval of immunotherapy for a variety of solid tumors including NSCLC, as a relatively immunogenic cancer species, relevant clinical researchs on SCLC are also underway and have made certain progress. More importantly, due to the existence of tumor heterogeneity, exploring relevant markers that can predict the efficacy of SCLC is essential for accurate therapy. This review describes the latest advances in SCLC immunotherapy and biomarkers related to the efficacy of immunotherapy.

小细胞肺癌（small cell lung cancer, SCLC）约占所有肺癌的15%，其具有高度侵袭性、倍增时间短、易早期转移且脑转移高发的特点，大约70%的患者初诊时已为广泛期^[1]^。治疗上，SCLC对放化疗敏感，近期客观缓解率（objective response rate, ORR）高但易复发转移，因此广泛期SCLC患者的5年生存率不到2%^[2]^。免疫疗法逐渐改变癌症治疗的格局，广义免疫治疗包括：免疫检查点抑制剂（immune checkpoint inhibitors, ICIs）、肿瘤疫苗、细胞因子治疗、免疫过继疗法等相关研究。近年来，免疫治疗被广泛研究及批准用于包括非小细胞肺癌在内的实体瘤，该疗法也开始在SCLC治疗中崭露头角。SCLC的发病与吸烟史强相关，且肿瘤突变负荷较高，具有潜在免疫源性，提示患者可能从免疫治疗中获益^[3]^。其次，有研究^[4]^表明合并副肿瘤综合征（尤其是肌无力综合征）的患者生存率更高，进一步佐证了免疫与SCLC预后潜在相关。2018年10月10日，ICIs联合化疗方案20年来首次改写SCLC美国国立综合癌症网络（National Comprehensive Cancer Network, NCCN）指南，然而，SCLC进展迅速的临床特征及易合并需要应用激素的上腔静脉综合征等^[5, 6]^，潜在地限制ICIs单药治疗的疗效^[7]^。因此，ICIs联合其他药物治疗成为现在临床研究的一个方向。本综述汇总了近5年以来SCLC免疫治疗取得的最新进展，并回顾了与疗效和预后相关的潜在标志物的研究，探讨了未来SCLC的免疫治疗方式及研究方向。

## 1 SCLC的免疫治疗进展

### 1.1 ICIs

#### 1.1.1 一线治疗

2013年（NCT00527735）、2016年（NCT01450761）两项关于标准含铂化疗联合细胞毒性T淋巴细胞相关蛋白4（cytotoxic T lymphocyte associate antigen-4, CTLA-4）抗体伊匹木单抗一线治疗广泛期SCLC的临床研究均未能证实无进展生存期（progression-free survival, PFS）或总生存期（overall survival, OS）的获益^[8, 9]^，尽管研究未获得阳性结果，但其设计为后续联合治疗研究的探索打下基础。

目前在SCLC中获得阳性研究结果的ICIs大多为细胞程序性死亡-配体1（programmed cell death ligand-1, PD-L1）抑制剂。IMpower133是一项评估PD-L1抑制剂阿特珠单抗联合卡铂+依托泊苷一线治疗广泛期SCLC患者的有效性和安全性的随机双盲Ⅲ期临床试验^[10]^。这项研究达到了两个主要研究终点，试验组相比对照组（卡铂联合依托泊苷）延长了中位OS（12.3个月*vs* 10.3个月，HR=0.70，*P*=0.006, 9）和中位PFS（5.2个月*vs* 4.3个月，HR=0.77，*P*=0.017）。基于该研究，2019年3月美国食品药品监督管理局（Food and Drug Administration, FDA）批准阿特珠单抗联合卡铂+依托泊苷方案用于广泛期SCLC患者的一线治疗。

CASPIAN研究^[11]^是另一项随机分组、开放标签的Ⅲ期临床研究，研究纳入未经治疗的广泛期SCLC患者，随机分为三组，分别是作为试验组的PD-L1抑制剂德瓦鲁单抗联合含铂化疗、德瓦鲁单抗+曲美母单抗（CTLA-4抑制剂）联合含铂化疗和作为对照组的标准含铂化疗（依托泊苷联合顺铂，EP方案）。结果显示，德瓦鲁单抗联合含铂化疗组的中位OS为13个月，而EP组为10.3个月（HR=0.73, *P*=0.004, 7）。远期生存数据显示，接受德瓦鲁单抗联合组的患者18个月生存率为33.9%，对照组只有24.7%。这证明EP方案联合德瓦鲁单抗用于治疗广泛期SCLC一线治疗可以显著提高总体存生存率（死亡风险降低27%）。

这两项Ⅲ期前瞻性随机对照研究确立了ICIs联合化疗在SCLC一线治疗领域的优势地位。另外，目前尚有多个探索ICIs联合含铂标准化疗一线治疗广泛期SCLC的研究正在进行（表 1）。然而与非小细胞肺癌相比，与ICIs联合的治疗方案给SCLC患者带来的获益仍有待提高。

**1:** 正在进行的SCLC免疫治疗相关临床研究（来源: clinicaltrials.gov, 最后更新: 2019.12.25） Ongoing immunotherapy trials in SCLC (source: clinicaltrials.gov, last accessed: 25^th^ December 2019)

Study	Arm	Primary endpoint	Design	Stage
First line				
NCT03066778	Pembro+ EP/Placebo+EP	PFS/OS	Phase 3, randomized, double-blind	ES-SCLC
NCT03811002	Chemoradiation/Chemoradiation+Atezo	PFS/OS	Phase 2/3, randomized	LS-SCLC
PAVE (NCT03568097)	Ave+EP	1-year PFS rate	Phase 2, single arm	ES-SCLC
NCT03382561	Nivo+EC/EC	PFS	Phase 2, randomized, controlled	ES-SCLC
JAVELIN Medley (NCT02554812)	Ave+Utomilumab	DLT/ORR	Phace 1b/2, randomized	ES-SCLC
NCT03406715	Ipi+Nivo+Dendritic cell based p53 vaccine	DCR	Phase 2, single arm	ES-SCLC
NCT03041311	Trilaciclib+Etoposide or Carboplatin or Atezo/ Placebo+Etoposide or Carboplatin or Atezo	OS/AE	Phase 2, randomized, double-blinded	ES-SCLC
Maintenance				
STIMULI (NCT02046733）	Nivo+Ipi/Observation	PFS/OS	Phase 2, randomized	LS-SCLC
ADRIATIC (NCT03703297）	Durva+Placebo/Durva+Treme/Placebo+Placebo	PFS/OS	Phase 3, randomized, double-blind	LS-SCLC
NCT03410368	Autologous natural killer cells/No intervention	PFS	Phase 2, randomized, controlled	ES-SCLC
IMPULSE (NCT03568097)	MGN1703/Continous first line therapy	OS	Phase 2, randomized	ES-SCLC
ACHILES (NCT03540420）	Atezo/Observation	2-year survival	Phase 2, randomized	LS-SCLC
NCT03585998	Durva	PFS	Phase 2, single arm	LS-SCLC
NCT03554473	M7824/M7824+Topotecan/M7824+Temozolomide	Efficacy	Phase 2, non-randomized	ES-SCLC
Second line or beyond				
NCT03059667	Atezo/Topotecan or EC	ORR	Phase 2, randomized, non-comparative	ES-SCLC
NCT03811379	Toripalimab	ORR	Phase 2, single arm	ES-SCLC
NCT03761914	Galinpepimut-S+Pembro	ORR	Phase 1/2, non-comparative, multi-arm	ES-SCLC
NCT03228667	ALT-803+Pembro/Nivo/Atezo/Ave	ORR	Phase 2b, non-randomized, single arm	ES-SCLC
NCT03575793	Nivol+Ipi/Plinabulin+Nivo+Ipi	MTD/PFS	Phase 1/2, randomized	ES-SCLC
NCT03728361	Nivol+Temozolomide	ORR	Phase 2, single arm	ES-SCLC
NCT03994744	Sintilimab+Metformin	ORR/Safety	Phase 2, single arm	ES-SCLC
NCT04192682	Anlotinib+Sintilimab	PFS	Phase 2/3, single arm	ES-SCLC
NCT04055792	Sintilimab+Anlotinib/Anlotinib	PFS	Phase 2, randomized, controlled	ES-SCLC
NCT03093688	Infusion of iNKT cells and CD8+ T cells	irAEs/ORR	Phase 1/2, single arm	ES-SCLC
SCLC: small cell lung cancer; ES-SCLC: extensive stage SCLC; LS-SCLC: limited-stage SCLC; Ipi: Ipilimumab; Durva: Durvalumab; Treme: Tremelimumab; Atezo: Atezolizumab; Nivo: Nivolumab; Pembro: Pembrolizumab; Ave: Avelumab; DCR: disease control rate; ORR: objective response rate; OS: overall survival; PFS: progression-free survival; DLT: dose-limiting toxicities; MTD: maximum tolerated dose; irAEs: immune-related adverse events.				

#### 1.1.2 维持治疗

尽管SCLC对化疗敏感，但容易短期内再次复发转移，因此一线治疗后的维持治疗一直是SCLC的探索热点，采用化疗药物和多靶点药物的维持治疗均以失败告终，因此，采用免疫治疗作为维持治疗的手段在临床上进行了初步探索，但结果均令人失望。一项Ⅱ期单臂试验评估了45例广泛期SCLC患者在EP方案化疗有效或病情稳定后接受细胞程序性死亡-1（programmed cell death-1, PD-1）抑制剂帕博利珠单抗维持治疗的疗效^[12]^，主要终点是中位PFS延长至3个月（比历史对照的2个月增加50%），但该队列中位PFS仅为1.4个月。

在另一项Ⅲ期试验Check-Mate 451（NCT02538666）研究中，经一线含铂化疗后疾病控制的广泛期SCLC患者被随机分为三组，一组接受PD-1抑制剂纳武利尤单抗单药治疗；一组接受纳武利尤单抗+伊匹木单抗联合治疗至4个周期，随后接受纳武利尤单抗单药治疗；一组使用安慰剂治疗（对照组），直到疾病进展或不可接受的毒性，最长治疗时间2年。该研究主要终点为，与对照组相比，使用ICIs组合治疗的患者OS得到改善，但最终并未达到研究终点。纳武利尤单抗+伊匹木单抗组的中位OS为9.2个月，含铂化疗组为9.6个月，免疫维持治疗并未改善患者的生存（HR=0.92, *P*=0.369, 3）。未来进一步的研究可能有助于确定从ICIs维持治疗获益的患者人群。此外，还有一些关于局限期SCLC放化疗后联合ICIs维持治疗的研究正在进行中（表 1）。

#### 1.1.3 二线及二线以上的治疗

Checkmate 032^[13]^是第一个评价免疫治疗用于一线铂类化疗失败的SCLC患者的试验，在这项Ⅰ期/Ⅱ期开放标签的临床试验中，入组了既往接受过2线及以上化疗的患者216例，随机分为单用纳武利尤单抗、纳武利尤单抗1 mg/kg+伊匹木单抗3 mg/kg、纳武利尤单抗3 mg/kg+伊匹木单抗1 mg/kg三组，研究结果显示主要终点客观缓解率（objective response rate, ORR）分别为10%、23%和19%。此外，三组中位PFS分别为1.4个月、2.6个月和1.4个月，中位OS分别为4.4个月、7.7个月和6.0个月。而2年中持续反应的三组患者中，单用纳武利尤单抗治疗的患者占45%，联合治疗的患者占36%，并且单药组的中位缓解有效时间（duration of response, DOR）为17.9个月，较纳武利尤单抗1 mg/kg+伊匹木单抗3 mg/kg组的14.2个月延长了3.7个月。有研究^[14]^提示纳武利尤单抗单药使用对患者的远期疗效更有优势，而且药物相关的毒性是可控的。在此研究结果的基础上，FDA批准了纳武利尤单抗用于SCLC晚期二线或者以上治疗。此外，一项比较阿特珠单抗对比依托泊苷+卡铂或拓扑替康作为广泛期SCLC二线治疗的疗效的Ⅱ期开放标签、随机非对照的临床研究NCT03059667，目前正在进行中。类似的评估也正在另外一项试验（NCT03811379）中进行（表 1）。

Ib期篮子研究Keynote-028^[15]^和Ⅱ期篮子研究Keynote-158^[16]^对帕博利珠单抗在晚期SCLC患者中的抗肿瘤活性进行了评估。两项试验共纳入131例SCLC患者，研究者对83例接受过≥2次治疗的患者进行了疗效分析，汇总分析^[17]^结果显示，ORR为19.3%（16/83），9例受试者（9/16, 61%）的DOR≥18个月，随访至24个月时，PFS和OS率分别为13%和21%，治疗相关的毒性反应与先前关于PD-1抑制剂的安全性特征的报道一致。这说明帕博利珠单抗在部分接受过2线及以上的治疗的广泛期SCLC患者中显示出抗肿瘤活性，且反应持久，在获得客观缓解的患者中大多数缓解持续时间不少于18个月，且药物相关的毒性可控。基于这些数据，FDA已经批准帕博利珠单抗用于接受过2线及以上的治疗后出现疾病进展的晚期SCLC患者。

#### 1.1.4 其他ICIs联合治疗方案

在SCLC的治疗中，除了前文所述化疗+ICIs、ICIs联合的组合外（表 2），还有一些研究关注于联合其他非ICIs机制药物。研究^[18]^发现细胞周期蛋白依赖性激酶4/6（cyclin-dependent kinases 4/6, CDK4/6）抑制剂Trilaciclib能缓解骨髓抑制。一项阿特珠单抗+依托泊苷+卡铂±Trilaciclib一线治疗广泛期SCLC的Ⅱ期研究正在进行（NCT03041311）。另外，有研究^[19]^显示多聚ADP核糖聚合酶（poly ADP-ribose polymerase, PARP）能够上调PD-L1的表达，并可能进一步增强与癌症相关的免疫抑制。一项Ⅱ期临床试验NCT02484404展开了PARP抑制剂奥拉帕尼联合德瓦鲁单抗治疗SCLC的研究^[20]^，纳入了20例复发性SCLC患者，研究结果显示完全缓解率达到了10.5%，临床获益率达到了21.1%，PD-L1单抗与PARP抑制剂联合治疗值得期待。

**2:** SCLC免疫检查点抑制剂临床研究数据总结 Summary of completed clinical trials of immune checkpoint inhibitors in SCLC

Study	IMpower133 (NCT02763579)	CASPIAN (NCT03043872)	CheckMate451 (NCT02538666)	KEYNOTE-028 (NCT02054806)	KEYNOTE-158 (NCT02628067)	CheckMate032 (NCT01928394)
Design	Phase 3, randomized, double-blind	Phase 3, randomized, comparative	Phase 3, randomized, double-blind	Phase 1b, single arm	Phase 2, single arm	Phase 1/2, multicenter, multi-cohort
Arm	Atezo±EC	Durva+Treme+EP/ Durva+EP/EP	Nivo/Nivo+Ipi/ EP	Pembro	Pembro	Nivo±Ipi
Sample size	*n*=403Atezo+EC: 201Atezo: 202	*n*=805 Durva+Treme+EP: 268 Durva+EP: 268Durva+EP: 269	*n*=834 Nivo: 275 Nivo+Ipi: 280Placebo: 279	*n*=24	*n*=107	*n*=216 Nivo3: 98Nivo1+Ipi3: 61Nivo3+Ipi1: 54
Patient	Previously untreated ES-SCLC	Previously untreated ES-SCLC	Maintenance therapy in ES-SCLC	Progressed after chemotherapy ES-SCLC	Progressed after chemotherapy ES-SCLC	Progressed after chemotherapy SCLC
Primary endpoint	PFS OS	OS	OS	SafetyORR	ORR	ORR
Result	Atezo+EC *vs* Atezo mOS: 12.3 mo *vs* 10.3 momPFS: 5.2 mo *vs* 4.3 mo	Durva+EP *vs* EP mOS: 13 mo *vs* 10.3 momPFS: 5.1 mo *vs* 5.4 mo OS12: 34% *vs* 25%	Nivo+Ipi *vs* EP 9.2 mo *vs* 9.6 mo (*P*=0.369, 3)	ORR: 37.5%mPFS: 1.9 momOS: 9.7 momDoR: 9.0 mo	ORR: 18.7%mPFS: 2.0 momOS: 8.7 mo73% DoR≥12 mo	Nivo3 *vs* Nivo1+Ipi3 *vs* Nivo3+Ipi1 ORR: 10% *vs* 23% *vs* 19%
Toxicity	Atezo+EC *vs* AtezoGrade 3/4: 39.9% *vs* 24.5%	Durva+EP *vs* EP Grade 3/4: 62% *vs* 62%	Nivo *vs* Nivo+Ipi *vs* EP All grades: 52% *vs* 12% *vs* 8%	All grades: 66.7%	All grades: 59%	Nivo3 *vs* Nivo1+Ipi3 *vs* Nivo3+Ipi1 Grade 3/4: 13% *vs* 30% *vs* 19%
OS12: 12-month overall survival rate; mDoR: median duration of response; mPFS: median progression-free survival; mOS: median overall survival; EP: Etoposide+Platinum; EC: Etoposide+Carboplatin; Ipi1: Ipilimumab 1 mg/kg; Ipi3: Ipilimumab 3 mg/kg; Nivo1: Nivolumab 1 mg/kg; Nivo3: Nivolumab 3 mg/kg.						

### 1.2 疫苗

研究^[21]^发现，接受过铂类治疗的广泛期SCLC患者中，提前接种由腺病毒作为载体过表达野生型*p53*基因的树突状细胞，与后续化疗的临床反应密切相关。而最近的一项正在进行的Ⅱ期试验（NCT03406715），也将评估在纳武利尤单抗和伊匹木单抗联合治疗基础上，加入基于树突状细胞制备的p53疫苗是否会改善复发SCLC患者的预后。

### 1.3 免疫过继疗法

基于过去十年化疗和细胞因子诱导的杀伤（cytokine-induced killer, CIK）细胞相结合在治疗多种癌症中显示出良好的疗效，一项旨在评价化疗联合CIK细胞对比化疗治疗广泛期SCLC的回顾性研究^[22, 23]^显示，联合治疗组的PFS明显长于对照组，且输注CIK细胞后未见严重不良反应，这提示化疗联合CIK细胞免疫治疗在未来可能作为一种可行的方法让我们对其进一步探索。

### 1.4 共刺激受体

CD137是一种表达在激活的免疫细胞上的共刺激受体，可致细胞毒性T细胞和自然杀伤细胞活性增强，并触发抗肿瘤反应^[24]^。Utomilumab是一种针对CD137的激动性单克隆抗体，可以增强不同效应细胞的抗肿瘤活性^[25]^，一项评估Avelumab+Utomilumab一线治疗广泛期SCLC的Ⅰ期/Ⅱ期随机性开放性研究目前正在进行（NCT02554812）。

### 1.5 细胞因子

细胞因子能够直接刺激肿瘤部位的免疫效应器，影响免疫细胞活性，如：干扰素和白细胞介素。目前已有相关研究^[26, 27]^，并取得相关进展，但其在生存获益及毒性反应上仍有局限性。

## 2 SCLC免疫治疗预后相关标志物

### 2.1 PD-L1蛋白表达

Gadgeel等^[12]^研究了一线化疗后接受帕博利珠单抗作为维持治疗的患者基质细胞中PD-L1的表达。基质表面表达PD-L1的患者的中位PFS和中位OS较无表达的患者长，分别为6.5个月和1.3个月以及12.8个月和7.6个月。

Keynote-158^[16]^的SCLC队列探索性分析了PD-L1阳性细胞数（包括肿瘤细胞、淋巴细胞和巨噬细胞）占所有细胞总数的比率，即PD-L1联合评分（combined positive score, CPS）的预测潜力，结果显示PD-L1阳性（CPS≥1%）的患者应用帕博利珠单抗后有更好的ORR（35.7% *vs* 6%）、1年PFS率（28.5% *vs* 8.2%）和1年OS率（53.1% *vs* 30.7%）。这两项研究证明PD-L1表达的高低与广泛期SCLC患者帕博利珠单抗单药维持治疗的预后有相关性。

### 2.2 肿瘤突变负荷（tumor mutation burden, TMB）

Checkmate 032^[13]^中，研究者进一步分析了组织样本中TMB，发现27%的患者经全外显子测序（whole exome sequencing, WES）提示具有高TMB（≥248个）。在TMB高的患者中，使用纳武利尤单抗治疗的患者1年的总生存率为35%，使用纳武利尤单抗+伊匹木单抗的患者为62%；而低/中TMB（0个-142个和143个-247个）患者接受联合治疗的1年总生存率为20%-26%。说明具有较高TMB（定义为高于研究人群突变分布的上1/3）的患者从治疗中获得了更好的疗效，特别是在联合治疗应用时^[28]^。

### 2.3 免疫相关毒性反应（immune-related adverse event, irAE）

在2019年世界肺癌大会（World Conference on Lung Cancer, WCLC）上，有研究团队口头报告了一项最新研究成果，该研究回顾性分析了157例接受了至少一种PD-（L）1抑制剂或联合应用CTLA-4抑制剂治疗的SCLC患者。研究发现，发生了irAE的患者（65例）具有更好的ORR、更长的PFS和OS，且在治疗后6周、9周和12周后无进展的比例比未发生irAE的患者更高。这证明接受免疫治疗的SCLC患者中，早期irAE的发生与预后存在相关性。

### 2.4 肿瘤免疫微环境(tumor microenvironment, TME）

TME影响着肿瘤的发展、侵袭、转移和结局。一项关注SCLC细胞与其TME的研究^[29]^表明，SCLC细胞分泌的白介素-15（interleukin-15, IL-15）通过抑制CD4^+^T细胞增殖可导致患者预后不良。另一项研究^[30, 31]^分析了来自SCLC患者的活检组织中的FOXP3^+^细胞浸润，证明FOXP3^+^细胞的比例也是患者不良预后的独立指标^[29]^。此外，肿瘤相关的CD45^+^细胞的数量也被证明对OS率有预测作用。

### 2.5 自身免疫抗体

在将伊匹木单抗与卡铂+依托泊苷联合治疗的临床研究中，自身免疫抗体阳性的患者（抗SOX2、抗Hu、抗YO、抗VGCCA、抗VGPCA、抗核、抗中性粒细胞胞浆抗体）中位PFS明显延长，且有更高ORR^[32]^。这也证明自身免疫抗体对预后的预测有潜在的提示意义。

## 3 总结

如何改善SCLC的预后是临床面临的主要困境。免疫治疗具有独特的抗肿瘤治疗机制，目前在SCLC领域取得一定突破同时也面临着难题及瓶颈。目前研究结果一方面提示化疗联合ICIs治疗策略可行；同时，探索预后相关的标志物以筛选对治疗有反应的人群也十分关键。目前仍有许多值得期待的免疫检查点相关临床试验正在进行中。另外，对于其他免疫治疗的方式，还需要更多前瞻性随机对照研究的数据去验证有效性及安全性。如何筛选出从不同免疫治疗方案中获益的人群，仍值得探索。

## References

[1] Nicholson AG, Chansky K, Crowley J (2016). The international association for the study of lung cancer lung cancer staging project: proposals for the revision of the clinical and pathologic staging of small cell lung cancer in the forthcoming eighth edition of the TNM classification for lung cancer. J Thorac Oncol.

[2] Riaz SP, Lüchtenborg M, Coupland VH (2012). Trends in incidence of small cell lung cancer and all lung cancer. Lung Cancer.

[3] Alexandrov LB, Ju YS, Haase K (2016). Mutational signatures associated with tobacco smoking in human cancer. Science.

[4] Maddison P, Gozzard P, Grainge MJ (2017). Long-term survival in paraneoplastic Lambert-Eaton myasthenic syndrome. Neurology.

[5] Nakahara Y, Sasaki J, Fukui T (2018). The role of prophylactic cranial irradiation for patients with small-cell lung cancer. Jpn J Clin Oncol.

[6] Würschmidt F, Bünemann H, Heilmann HP (1995). Small cell lung cancer with and without superior vena cava syndrome: a multivariate analysis of prognostic factors in 408 cases. Int J Radiat Oncol Biol Phys.

[7] Arbour KC, Mezquita L, Long N (2018). Impact of baseline steroids on efficacy of programmed cell death-1 and programmed death-ligand 1 blockade in patients with non-small-cell lung cancer. J Clin Oncol.

[8] Reck M, Bondarenko I, Luft A (2013). Ipilimumab in combination with paclitaxel and carboplatin as first-line therapy in extensive-disease-small-cell lung cancer: results from a randomized, double-blind, multicenter phase 2 trial. Ann Oncol.

[9] Reck M, Luft A, Szczesna A (2016). Phase Ⅲ randomized trial of ipilimumab plus etoposide and platinum versus placebo plus etoposide and platinum in extensive-stage small-cell lung cancer. J Clin Oncol.

[10] Horn L, Mansfield AS, Szczęsna A (2018). First-line atezolizumab plus chemotherapy in extensive-stage small-cell lung cancer. N Engl J Med.

[11] Paz-Ares L, Dvorkin M, Chen Y (2019). Durvalumab plus platinum-etoposide versus platinum-etoposide in first-line treatment of extensive-stage small-cell lung cancer (CASPIAN): a randomised, controlled, open-label, phase 3 trial. Lancet.

[12] Gadgeel SM, Pennell NA, Fidler MJ (2018). Phase Ⅱ study of maintenance pembrolizumab in patients with extensive-stage small cell lung cancer (SCLC). J Thorac Oncol.

[13] Antonia SJ, López-Martin JA, Bendell J (2016). Nivolumab alone and nivolumab plus ipilimumab in recurrent small-cell lung cancer (CheckMate 032): a multicentre, open-label, phase 1/2 trial. Lancet Oncol.

[14] Pawel J, Jotte R, Spigel DR (2014). Randomized phase Ⅲ trial of amrubicin versus topotecan as second-line treatment for patients with small-cell lung cancer. J Clin Oncol.

[15] Ott PA, Elez E, Hiret S (2017). Pembrolizumab in patients with extensive-stage small-cell lung cancer: results from the phase Ib KEYNOTE-028 study. J Clin Oncol.

[16] Chung HC, Lopez-Martin JA, Kao SC (2018). Phase 2 study of pembrolizumab in advanced small-cell lung cancer (SCLC): KEYNOTE-158. Am Sco Clin Oncol.

[b17] 17Chung HC, Piha-Paul SA, Lopez-Martin J, et al. Pembrolizumab after two or more lines of previous therapy in patients with recurrent or metastatic small-cell lung cancer: results from the KEYNOTE-028 and KEYNOTE-158 studies. J Thorac Oncol, 2019. pii: S1556-0864(19)33850-X. doi: 10.1016/j.jtho.2019.12.109

[18] Weiss JM, Csoszi T, Maglakelidze M (2019). Myelopreservation with the CDK4/6 inhibitor trilaciclib in patients with small-cell lung cancer receiving first-line chemotherapy: a phase Ib/randomized phase Ⅱ trial. Ann Oncol.

[19] Jiao S, Xia W, Yamaguchi H (2017). PARP inhibitor upregulates PD-L1 expression and enhances cancer-associated immunosuppression. Clin Cancer Res.

[20] Thomas A, Vilimas R, Trindade C (2019). Durvalumab in combination with olaparib in patients with relapsed SCLC: Results from a phase Ⅱ study. J Thorac Oncol.

[21] Antonia SJ, Mirza N, Fricke I (2006). Combination of p53 cancer vaccine with chemotherapy in patients with extensive stage small cell lung cancer. Clin Cancer Res.

[22] Huang J, Kan Q, L an (2017). Chemotherapy in combination with cytokine-induced killer cell transfusion: an effective therapeutic option for patients with extensive stage small cell lung cancer. Int Immunopharmacol.

[23] Hontscha C, Borck Y, Zhou H (2011). Clinical trials on CIK cells: first report of the international registry on CIK cells (IRCC). J Cancer Res Clin Oncol.

[24] Sanchez-Paulete AR, Labiano S, Rodriguez-Ruiz ME (2016). Deciphering CD137 (4-1BB) signaling in T-cell costimulation for translation into successful cancer immunotherapy. Eur J Immunol.

[25] Segal NH, He AR, Doi T (2018). Phase I study of single-agent utomilumab (PF-05082566), a 4-1BB/CD137 agonist, in patients with advanced cancer. Clin Cancer Res.

[26] Zarogoulidis K, Ziogas E, Boutsikou E (2013). Immunomodifiers in combination with conventional chemotherapy in small cell lung cancer: a phase Ⅱ, randomized study. Drug Des Devel Ther.

[27] Pillai RN, Aisner J, Dahlberg SE (2014). Interferon alpha plus 13-cis-retinoic acid modulation of BCL-2 plus paclitaxel for recurrent small-cell lung cancer (SCLC): an Eastern Cooperative Oncology Group study (E6501). Cancer Chemother Pharmacol.

[28] Hellmann MD, Callahan MK, Awad MM (2018). Tumor mutational burden and efficacy of nivolumab monotherapy and in combination with ipilimumab in small-cell lung cancer. Cancer Cell.

[29] Wang W, Hodkinson P, McLaren F (2012). Small cell lung cancer tumour cells induce regulatory T lymphocytes, and patient survival correlates negatively with FOXP3^+^ cells in tumour infiltrate. Int J Cancer.

[30] Wang W, Hodkinson P, McLaren F (2013). Histologic assessment of tumor-associated CD45(+) cell numbers is an independent predictor of prognosis in small cell lung cancer. Chest.

[31] Bremnes RM, Sundstrom S, Aasebø U (2003). The value of prognostic factors in small cell lung cancer: results from a randomised multicenter study with minimum 5 year follow-up. Lung Cancer.

[32] Arriola E, Wheater M, Galea I (2016). Outcome and biomarker analysis from a multicenter phase 2 study of ipilimumab in combination with carboplatin and etoposide as first-line therapy for extensive-stage SCLC. J Thorac Oncol.

